# Pharmacoepidemiological Analysis of Oral Contraceptive Use in Adolescents in a German Longitudinal Cohort Study

**DOI:** 10.3390/children10020393

**Published:** 2023-02-16

**Authors:** Markus Herzig, Astrid Bertsche, Cornelia Hilbert, Wieland Kiess, Thilo Bertsche, Martina Patrizia Neininger

**Affiliations:** 1 Clinical Pharmacy, Institute of Pharmacy, Medical Faculty, Leipzig University, Brüderstraße 32, 04103 Leipzig, Germany; 2 Drug Safety Center, Leipzig University and University Hospital, Brüderstraße 32, 04103 Leipzig, Germany; 3 Center for Pediatric Research, University Hospital for Children and Adolescents, Liebigstraße 20a, 04103 Leipzig, Germany; 4 Division of Neuropediatrics, University Hospital for Children and Adolescents, Fleischmannstr. 8, 17475 Greifswald, Germany; 5 LIFE Leipzig Research Centre for Civilization Diseases, Leipzig University, Philipp-Rosenthal-Straße 27, 04103 Leipzig, Germany

**Keywords:** cohort study, pharmacoepidemiology, adolescent, pediatrics, contraceptive agents, blood pressure

## Abstract

To assess the use of oral contraceptives (OC) in adolescents, using data from a longitudinal, population-based pediatric cohort study (LIFE Child). We also investigated associations between OC use and socioeconomic status (SES), and associations between OC use and potential adverse drug reactions such as effects on blood pressure. We included 609 female participants of the LIFE Child cohort, aged ≥13 to <21 years, who visited the study center between 2012 and 2019. Data collection compromised drug use in the past 14 days, SES, and anthropometric data such as blood pressure. An analysis of covariance was used to detect potential associations between participants’ blood pressure and OC. Multivariate binary logistic regression was used to obtain odds ratios (aOR) adjusted for age and 95% confidence intervals (95% CI). The prevalence of OC use was 25.8%. OC intake was less common in participants with a high SES (aOR 0.30, 95% CI 0.15, 0.62). The mean age at OC initiation did not change between 2012 and 2019. We observed an increased use of second-generation OC (2013: 17.9%, 2019: 48.5%; *p* = 0.013) and a decreased use of fourth-generation OC (2013: 71.8%, 2019: 45.5%; *p* = 0.027). We found a higher systolic (mean: 111.74 mmHg, *p* < 0.001) and diastolic (69.15 mmHg, *p* = 0.004) blood pressure in OC users compared to non-users (systolic: 108.60 mmHg; diastolic: 67.24 mmHg). Every fourth adolescent took an OC. The share of second-generation OC increased during the study period. OC intake was associated with low SES. OC users had a slightly higher blood pressure than non-users.

## 1. Introduction

Oral contraceptives (OC) are one of the most important contraceptive options. According to a United Nations (UN) report from 2019, the frequency of OC use in the age group 15–49 years differs worldwide between 5.3% in Asia and 19.1% in Europe [1]. A study assessing the prevalence of preferred contraceptive methods in Latin America and the Caribbean countries showed that considerable differences in the same age group are noticeable even within a continent, such as short-acting reversible contraceptive methods being least preferred in Mexico (14%), and most preferred in Paraguay (54%) [2]. In general, the worldwide use of any contraceptive method in the age group 15–49 years increased by 8.5%, from 54.8% to 63.3%, between 1990 and 2010 [3]. However, most current data about OC use refer only to adults, and studies in adolescents are scarce. A study in the Scandinavian countries reported a prevalence of OC use of about 35% to 45% in the age group from 15 to 19 years [4]. Another study from Germany showed that every fifth adolescent took an OC between 2003 and 2006 [5]. However, these studies do not provide recent data, especially for Europe. In a recent study conducted in Brazil, the prevalence of OC use was considerably higher than in Europe, with 62% at the age of 15 years and 58% at the age of 18 years [6]. Furthermore, it is still unclear whether socioeconomic factors such as household income or educational status are associated with the use of OC [7,8].

However, hormonal preparations used for contraception can also be used for other indications, e.g., acne and dysmenorrhea [9]. Especially in the case of acne, OC can lead to a better skin appearance [10]. Nevertheless, OC are also heavily criticized, especially regarding possible adverse drug reactions (ADRs). An association between an increased risk of venous thromboembolism and the intake of OC, especially the newer generations of active ingredients, has been established [11,12]. This ADR has led to a change in prescribing behavior, with an increased preference for older generations of active ingredients. In an Irish study conducted between 2008 and 2013, an increase in the prescribing of second-generation combined OC and a decrease in the prescribing of third- and fourth-generation combined OC were observed in adolescent females aged 16 to 24 years [13]. However, these data do not reflect recent years, indicating an interest in providing more current data on possible trends.

Furthermore, the discussion about ADRs includes the potential increase in blood pressure, as some studies found an association between increased blood pressure and OC use, whereas other studies could not confirm this association [5,14,15,16,17,18,19,20]. In addition, according to Cochrane reviews, there is only limited evidence for body weight gain in relation to OC use [21,22]. Further investigation of a possible impact of OC use on body weight is of particular interest, especially in adolescents, as high levels of anxiety regarding body weight gain in female adolescents were reported [23]. This underlines the importance of epidemiological studies allowing the observation of potential ADRs in large cohorts, especially since ADRs in routine pediatric care are underreported [24].

Therefore, we conducted a pharmacoepidemiological investigation to determine the prevalence and associated factors of OC use in adolescents, to ascertain possible trends in OC generations used during the study period, and to investigate possible ADRs of OC, such as effects on blood pressure or body weight.

## 2. Materials and Methods

### 2.1. Study Design

We evaluated data obtained in the ‘Leipzig Research Centre for Civilization Diseases (LIFE) Child study’, which is an ongoing longitudinal population-based cohort study. The research center is located at the University Hospital of Leipzig (Saxony, Germany). The Ethics Committee of Leipzig University approved the study protocol [25]. The recruitment of the participants included different locations and communication channels throughout the study period [25,26]. For follow-up examinations, participants were invited to the study center annually until their 21st birthday. The end of participant observation was set to the child’s 21st birthday in accordance with the recommendations of the American Academy of Pediatrics, the U.S. Department of Health, and the Food and Drug Administration, as current studies do not assume a completed physical development for the age groups below [27].

Written informed consent was obtained from participating adolescents and their parents. More information about the study design, data collection, and other details has been published previously [25,26,28].

### 2.2. Inclusion Criteria

The LIFE Child study started in 2011. We evaluated the data from 1 January 2012 to 31 December 2019, since continuous, consistent data quality for OC use was available from 2012 onwards, and data collection in 2020/2021 was limited due to the SARS-CoV-2 pandemic. As we observed in a preliminary analysis that the first use of an OC occurred at the age of 13 years, we included participants from the age of 13 years onwards.

### 2.3. Data Collection

All data were re-recorded at each visit. Using a structured questionnaire, study assistants documented all drugs taken in the 14 days prior to the visit to the study center. Participants were asked to bring the drugs to the study center. Missing information was collected in a phone call after the visit to the study center. Participants and the persons who accompanied the adolescents were asked to bring the drugs taken to the study center. Those drugs were scanned and documented via a unique code or documented manually.

Data collection included additional anthropometric data, e.g., body weight and height, as well as the current systolic and diastolic blood pressure.

The socioeconomic status (SES) was also part of the data collection and was measured using the “Winkler-Stolzenberg Index”, which is classified into three categories: low (3–8.4 points), medium (8.5–15.4 points), and high (15.5–21.0 points). The calculation compromises parental education, occupational status, and household equivalized disposable income [29].

### 2.4. Data Processing

The last visit of each participant in the study period was used to calculate prevalences and associations to avoid biases possibly resulting from multiple visits and to ensure a distribution of participants across all age groups, as the first visit would over-represent younger age groups. However, in order to detect changes over the study years at the level of active ingredients, the entire longitudinal dataset with all recorded visits was used for the analyses of the active ingredients.

We evaluated all oral drugs belonging to the Anatomical Therapeutic Chemical classification (ATC) group “Hormonal Contraceptives for Systemic Use” (ATC code: G03A) and drugs containing the combination of cyproterone and estrogen (ATC code: G03HB01), as they can also be used for contraception. We classified the recorded preparations into generations based on the progestins contained, using a classification system adapted from the French system [30]: First-generation compromised norethisterone, second-generation levonorgestrel, third-generation desogestrel, and fourth-generation dienogest, drospirenone, chlormadinone, nomegestrol, and cyproterone.

Additionally, we used anthropometric data such as body weight and height, current systolic and diastolic blood pressure, and SES in our analyses.

### 2.5. Statistical Analysis

We used IBM SPSS Statistics 28.0.1.1 (IBM, Armonk, NY, USA). To investigate possible changes in the intake of the different generations of OC between 2013 and 2019, chi-square tests with Yates’ correction were performed.

Prevalences were calculated as the percentage of participants reporting OC use in the past 14 days. The calculations were accomplished by bootstrap calculations (replications: 2000; bias-corrected) to obtain 95% confidence intervals (95% CI). Multivariate binary logistic regressions adjusted for age and SES were performed to calculate the adjusted odds ratio (aOR) with 95% CI for OC use. We controlled the respective variables for multicollinearity. Since the correlation coefficient was <0.85, the variables were included in the model [31]. If a case in the data set had a missing value in one of the respective variables, it was excluded from the analysis.

To determine the effects of OC use on systolic and diastolic blood pressure, we performed an analysis of covariance (ANCOVA) and an analysis of variance (ANOVA). These different methods were used to detect effects across the entire study population (ANCOVA), and to control for effects of age, body height, and socioeconomic factors (ANOVA). For the ANCOVA and ANOVA, participants who could not specify their OC preparation or who used progestin mono-preparations were excluded, since the potential effect on blood pressure is attributed to the estrogens [32]. Participants using non-oral contraceptives were excluded from these analyses, owing to the potential to bias the results. For the ANCOVA, the model was adjusted for the covariates of age and body height. Both models fulfilled the requirements of variance homogeneity and normal distribution; the ANCOVA model additionally fulfilled the homogeneity of the covariates.

For the ANOVA, we performed propensity score matching (SPSS-Extension: STATS_PSM, Version: 2.0.1; match-tolerance = 0.25) to select appropriate non-users who reported no OC use at their last visit and who matched as closely as possible in age, body height, and SES to the users of OC at their last visit. This matching resulted in 111 participants in each group. The matched groups were also used to identify possible effects of OC use on body weight using a Mann–Whitney-U test, owing to the non-normal distribution of the data on body weight.

The results of ANOVA and ANCOVA are reported with an F-value (ratio of two variances), an F-distribution (degrees of freedom), *p*-values, and a partial η^2^ (effect size). The effect size according to Cohen’s classification was categorized as small effect (η_p_^2^ ≥ 0.01), medium effect (η_p_^2^ ≥ 0.06), and large effect (η_p_^2^ ≥ 0.14) [33]. A *p*-value of ≤0.05 was considered to indicate significance.

## 3. Results

### 3.1. Characteristics of the Study Population

In our evaluation, we included the last visit of 609 adolescents visiting the study center (Table 1). The median age at the participants’ last visit was 16.0 years (Q25/Q75: 14.6/17.5, min./max.: 13.0/20.5). As shown in Table 1, 50.6% of the participants were allocated to medium SES. A total of 157 participants who used OC were recorded.

### 3.2. Prevalence of OC Use

The total prevalence was 25.8% (95% CI 22.5%, 29.1%). The number of adolescents taking an OC in the past 14 days increased with age (Table 2). A high SES was associated with a lower prevalence of OC use compared to a low SES (aOR 0.30, 95% CI 0.15, 0.62).

### 3.3. Median Age of Onset of OC Use

**Figure 1:**
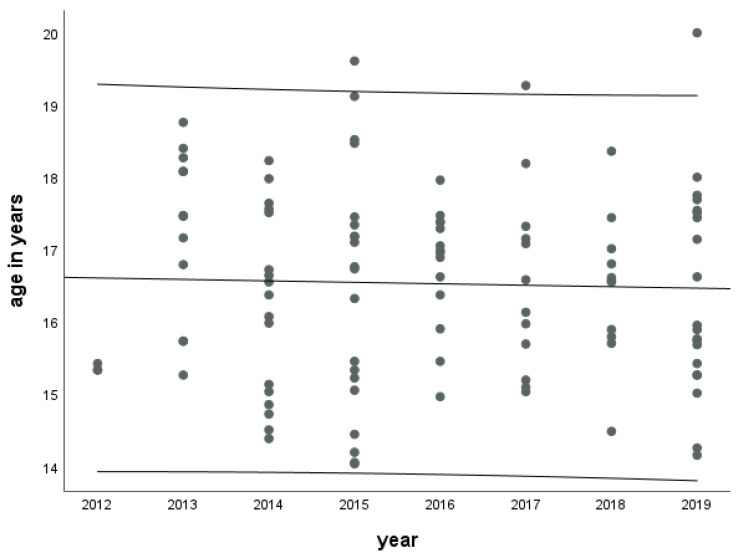
Age at initiation of oral contraceptive use during the study period by study year (number of affected participants = 109). The figure shows the regression line with a linear slope and associated 95% confidence intervals. LIFE Child 2012–2019.

Between 2012 and 2019, 109/609 (17.9%) participants initiated the use of OC. Another 48/609 (7.9%) of participants had already taken OC at the first study appointment, and thus the initiation could not be determined. The median age at the initiation of OC was 16.6 years (Q25/Q75: 15.4/17.5, min./max.: 14.1/20.0) in those who initiated the use during the study period. The age at initiation did not change between 2012 and 2019 (Figure 1).

### 3.4. Changes in Active Ingredients of OC

A total of 293 OC were recorded at all recorded visits. The most frequently reported combination between 2012 and 2019 was dienogest/ethinylestradiol (36.9%, Table 3). The second generation of OC constituted 17.9% of all reported active ingredients in 2013 and 48.5% in 2019 (*p* = 0.013). In contrast, the fourth generation of OC constituted 71.8% of all reported active ingredients in 2013, reached a maximum of 86.0% in 2014, and represented 45.5% in 2019 (2013 to 2019: *p* = 0.027). Owing to 25% of the values for the precise active ingredient being missing in 2012, we did not include that year in the calculations.

### 3.5. Associations between OC Use and Blood Pressure

As displayed in Table 4, the ANCOVA using data from the whole study population showed that OC users had a slightly higher systolic and diastolic blood pressure than non-users. The ANOVA confirmed this small effect on systolic and diastolic blood pressure between OC users and non-users according to Cohen’s classification.

### 3.6. Body Weight and OC Use

Using a Mann–Whitney-U test, no differences were found between the user group (median: 60.5 kg, Q25/Q75: 54.9/69.5, min./max.: 42.5/117.8, *p* = 0.259) and the non-user group (median: 59.2 kg, Q25/Q75: 54.4/67.8, min./max.: 44.2/128.0) in terms of body weight.

## 4. Discussion

This evaluation of a longitudinal pediatric cohort study showed that every fourth female participant used an OC. The age group of 19-year-old females used OC most frequently, at almost 50%. Low SES was associated with increased use of OC. We further observed an increase in the intake of second-generation OC during the study period. Additionally, we found that OC users had a slightly higher systolic and diastolic blood pressure than non-users. However, OC use was not associated with a higher body weight.

### 4.1. Prevalence of OC use

In our study population, we observed that every fourth adolescent participant used an OC. A global overview of different contraceptive methods and their prevalences for the age group 15–49 years is provided by a 2019 UN report [1]. In this report, the prevalence for OC use in Germany was given as 32%. In general, the highest prevalences of OC use were reported for countries on the continents of Europe (e.g., the Czech Republic: 34.4%), North America (e.g., Canada: 28.5%; USA: 13.7%), and Australia (22.0%), as part of Oceania. Low prevalences were reported for various countries in Oceania (e.g., Tonga: 1.2%), Asia (e.g., Japan: 2.9%), and Africa (e.g., Ethiopia: 1.4%). Thus, the frequency of OC use varies between continents and countries worldwide, and Germany is one of the countries with the highest rate of OC use. On the other hand, the report shows that many other countries use other contraceptive methods instead of OC. To give an example, in South Africa, only 5% of females use OC, whereas 23% use injectables and 12% use male condoms. In contrast, in some countries, the use of contraceptives is generally rare and unsafe methods, such as the rhythm or withdrawal method, are used. For instance, a high rate of unsafe contraceptive methods is reported for Peru, where 8% of females use the rhythm method and 5% of females use the withdrawal method [1]. Concurrently, 60% of pregnancies in Peru are reported to be unintended [34]. The WHO study that determined this value evaluated data from 36 low- and middle-income countries and detected that 56% of unintended pregnancies resulted from not using any contraceptive method [34].

A limitation of the 2019 UN report is that an age classification was not provided and that it was limited to the age group 15–49 years [1]. Additionally, the 2019 UN report is based on estimates because data on the prevalences of OC use among adolescents are scarce.

A study conducted in Scandinavian countries (Denmark, Norway, and Sweden) reported prevalences for combined OC use ranging from approximately 35% to 45% in the age group 15–19 years in 2013 [4]. In contrast, we observed a much lower prevalence of 20% in 15-year-olds, which indicates a different intake behavior in Germany compared to other countries, even within Europe. This is supported by another study from Germany, which reported a similar prevalence of 15% for 15-year-olds and a prevalence of 20% for the age group 13–17 years between 2003 and 2006 [5].

### 4.2. OC Use and SES

We found an association between OC intake and low SES. In contrast, a US study observed an increased use of contraceptives among adult women with a college education or higher [8]. In adolescents, on the other hand, contraceptive use also depends on the initiation of sexual activity, which begins earlier if household wealth and/or maternal college education is lower [35]. Furthermore, health insurance in Germany, as in many other countries, covers the costs of OC use in adolescents and young adults [36]. This may lead to a different result compared to the studies conducted with adults, as the affordability of an OC may play only a minor role in young people’s decision for or against an OC.

### 4.3. Changes in Generations of OC Used between 2013 and 2019

We observed an increase in the use of second-generation OC and a decrease in the use of fourth-generation OC between 2013 and 2019. Debates about the potential ADRs of OC use may have contributed to this change. For example, venous thromboembolism risk was significantly increased with cyproterone acetate, desogestrel, drospirenone, and gestodene (third and fourth generations of OC) compared to levonorgestrel in systematic reviews from 2014 and 2017 [11,12]. Disputes between politicians and the European Medicines Agency may have contributed to the increased public interest and pushed awareness of this ADR into the general public [37]. In another study, the authors concluded that a controversial discussion about these ADRs resulted in lower prescription rates for third- and fourth-generation OC in several European countries [13].

### 4.4. Associations between OC Use and Blood Pressure

The possible influence of OC on blood pressure has been discussed controversially for decades. Several studies have observed an increase in blood pressure in participants taking OC [5,17,18,19]. We also overserved these effects in our study. A 2018 review concluded that exogenous estrogen, even at low concentrations, can activate the renin–angiotensin–aldosterone system, leading to water and sodium retention and resulting in increased blood pressure [32]. Thus, plausible physiological mechanisms are known that can support the argument for an increase in blood pressure. However, a number of studies could not reveal such an effect, and the authors of these studies concluded that OC had no effect on blood pressure [14,15,16,20]. As we performed an epidemiologic study in which actual self-reported drug use and real-life anthropometric data were recorded, we detected possible associations that may not be represented by these studies. Regardless of the results of the studies mentioned, it is questionable whether a small increase in systolic and diastolic blood pressure in healthy females is always clinically relevant. For females with elevated blood pressure, the increase may be relevant to avoid an accumulation of risk factors. Therefore, both the WHO guideline and the guideline of the “German Society of Gynaecology and Obstetrics” do not recommend the use of OC in patients with hypertension [38,39].

### 4.5. Associations with Body Weight Gain

In our study, we did not find an association between OC use and body weight gain. According to Cochrane reviews, an association between body weight gain and the intake of combined oral contraceptives is not evident [21,22]. These Cochrane reviews criticize the analyzed studies for their disregard for natural body weight gain with age, especially in adolescence. To address this issue, we matched OC users and non-users according to body height and age to eliminate the influence of these factors on body weight in our calculation. This allows us to support the conclusions of the Cochrane reviews, as we found no differences in body weight between these matched groups.

### 4.6. Limitations

As in other cohort studies [40,41,42], the LIFE Child study included more participants with middle and high SES. To minimize this bias, LIFE Child intensified its efforts to recruit as evenly as possible across all social milieus by recruiting entire school classes [25,26]. In addition, the higher participation rate of participants with middle and high SES was considered in the calculations of the adjusted odds ratios.

A further bias may result from the fact that not all participants attended all possible appointments between 2012 and 2019. This problem is common in longitudinal cohort studies owing to factors such as low educational status and low household income [43,44].

As OC are sometimes prescribed for several reasons at the same time, e.g., to improve skin appearance or relieve dysmenorrhea in addition to contraception, we cannot exclude that OC preparations were taken for other reasons than contraception.

### 4.7. Conclusions

Our evaluation shows that every fourth female adolescent used an OC. The intake was associated with a low SES. During the study period between 2013 and 2019, we observed a decrease in the use of fourth-generation OC and an increase in second-generation OC. Furthermore, we did not find an association between OC use and body weight, but we did find that OC users had a slightly higher systolic and diastolic blood pressure than non-users. Since the additional increase in blood pressure may be harmful to the progression of disease in patients with known cardiovascular risk factors and/or hypertension, these effects on blood pressure should be considered in treatment decisions.

## Figures and Tables

**Table 1:** Sociodemographic characteristics of participants between 2012 and 2019. The data presented in the table were collected at participants’ last visit during the study period. LIFE Child 2012–2019.

	n	%
**Total**	609	100.0
**Age group**		
13	93	15.3
14	87	14.3
15	128	21.0
16	98	16.1
17	97	15.9
18	63	10.3
19	25	4.1
20	18	3.0
**SES ^a^**		
Low	69	11.3
Middle	308	50.6
High	139	22.8
Unknown	93	15.3
**Monthly household equivalent income ^b^**		
EUR ≤ 787	79	13.0
EUR 788–999	58	9.5
EUR 1000–1190	73	12.0
EUR 1191–1400	62	10.2
EUR 1401–1667	64	10.5
EUR ≥ 1668	175	28.7
Unknown	98	16.1
**Highest educational status of parents**		
No school certificate	3	0.5
Lower secondary education	43	7.1
Middle secondary education	165	27.1
Higher secondary education	285	46.8
Other	11	1.8
Unknown	102	16.7
**Highest occupational status of parents**		
Unemployed	19	3.1
Worker, employed, civil service	280	46.0
Self-employed	85	14.0
Other	21	3.4
Unknown	204	33.5

^a^ Socioeconomic status ^b^ The monthly net equivalent household income takes into account the number of family members in the household.

**Table 2:** Prevalence of oral contraceptive (OC) use in adolescents. Multivariate binary logistic regressions were used to determine adjusted odds ratios (aOR) for age (adjusted for socioeconomic status [SES]) and SES (adjusted for age). The data shown refer to the last visit of the participants. LIFE Child 2012–2019.

	n	Prevalence in % (95% CI)	aOR (95% CI)	*p*-Value
**Total**	157/609	25.8 (22.5, 29.1)	-	-
**Age**				
13	1/93	1.1 (0.0, 3.2)	0.09 (0.01, 0.71)	0.022
14	11/87	12.6 (6.9, 18.4)	Reference	
15	26/128	20.3 (14.1, 26.6)	1.98 (0.89, 4.40)	0.096
16	28/98	28.6 (20.4, 36.7)	3.20 (1.42, 7.18)	0.005
17	42/97	43.3 (35.1, 51.5)	6.85 (3.06, 15.31)	<0.001
18	29/63	46.0 (34.9, 57.1)	6.05 (2.44, 15.04)	<0.001
19	13/25	52.0 (36.0, 68.0)	7.25 (1.98, 26.52)	0.003
20	7/18	38.9 (22.2, 55.6)	6.78 (1.29, 35.63)	0.024
**SES**				
Low	25/69	36.2 (26.1, 46.4)	Reference	
Middle	79/308	25.6 (21.1, 30.2)	0.47 (0.26, 0.86)	0.014
High	24/139	17.3 (12.2, 22.3)	0.30 (0.15, 0.62)	0.001

**Table 3:** Frequency of active ingredients in the reported OC (total number of oral contraceptives = 293) by study year. The data shown refer to all recorded visits of the participants. LIFE Child 2012–2019.

		ATC-Classification	2012 [n/n_year_^a^ (%)]	2013 [n/n_year_^a^ (%)]	2014 [n/n_year_^a^ (%)]	2015 [n/n_year_^a^ (%)]	2016 [n/n_year_^a^ (%)]	2017 [n/n_year_^a^ (%)]	2018 [n/n_year_^a^ (%)]	2019 [n/n_year_^a^ (%)]	Frequency [n/n_total_ (%)]
**Progestin**	**Estrogen**										
Dienogest	Ethinylestradiol	G03AA16	9/28 (32.1)	13/39 (33.3)	18/50 (36.0)	18/48 (37.5)	16/44 (36.4)	13/30 (43.3)	10/21 (47.6)	11/33 (33.3)	108/293 (36.9)
Levonorgestrel	Ethinylestradiol	G03AA07	4/28 (14.3)	7/39 (17.9)	5/50 (10.0)	7/48 (14.6)	10/44 (22.7)	7/30 (23.3)	5/21 (23.8)	16/33 (48.5)	61/293 (20.8)
Chlormadinone	Ethinylestradiol	G03AA15/G03AB07	2/28 (7.1)	9/39 (23.1)	16/50 (32.0)	13/48 (27.1)	11/44 (25.0)	5/30 (16.7)	3/21 (14.3)	2/33 (6.1)	61/293 (20.8)
Drospirenone	Ethinylestradiol	G03AA12	5/28 (17.9)	3/39 (7.7)	4/50 (8.0)	3/48 (6.3)	-	1/30 (3.3)	1/21 (4.8)	1/33 (3.0)	18/293 (6.1)
Desogestrel		G03AC09	-	1/39 (2.6)	-	3/48 (6.3)	4/44 (9.1)	2/30 (6.7)	1/21 (4.8)	1/33 (3.0)	12/293 (4.1)
Nomegestrol	Estradiol	G03AA14	-	1/39 (2.6)	3/50 (6.0)	2/48 (4.2)	1/44 (2.3)	1/30 (3.3)	1/21 (4.8)	-	9/293 (3.1)
Cyproterone	Estrogen	G03HB01	1/28 (3.6)	2/39 (5.1)	2/50 (4.0)	1/48 (2.1)	1/44 (2.3)	-	-	1/33 (3.0)	8/293 (2.7)
Desogestrel	Ethinylestradiol	G03AA09	-	1/39 (2.6)	-	-	1/44 (2.3)	1/30 (3.3)	-	-	3/293 (1.0)
Unknown active ingredient		G03xxxx	7/28 (25.0)	2/39 (5.1)	2/50 (4.0)	1/48 (2.1)	-	-	-	1/33 (3.0)	13/293 (4.4)
Second generation ^b^		-	4/28 (14.3)	7/39 (17.9)	5/50 (10.0)	7/48 (14.6)	10/44 (22.7)	7/30 (23.3)	5/21 (23.8)	16/33 (48.5)	61/293 (20.8)
Third generation ^c^		-	-	2/39 (5.1)	-	3/48 (6.3)	5/44 (11.4)	3/30 (10.0)	1/21 (4.8)	1/33 (3.0)	15/293 (5.1)
Fourth generation ^d^		-	17/28 (60.7)	28/39 (71.8)	43/50 (86.0)	37/48 (77.1)	29/44 (65.9)	20/30 (66.7)	15/21 (71.4)	15/33 (45.5)	204/293 (69.6)

^a^ Total number of oral contraceptives reported in the given year; ^b^ second generation comprised levonorgestrel; ^c^ third generation comprised desogestrel; ^d^ fourth generation comprised dienogest, drospirenone, chlormadinone, nomegestrol, and cyproterone.

**Table 4:** Results of analysis of covariance (ANCOVA) and analysis of variance (ANOVA) on differences in systolic and diastolic blood pressure between users of OC and non-users. LIFE Child 2012–2019.

	ANCOVA ^a^					ANOVA ^b^				
	Non-Users of OC		Users of OC		Result ANCOVA	Non-Users of OC		Users of OC		Result ANOVA
	Mean	SD ^c^	Mean	SD ^c^		Mean	SD ^c^	Mean	SD ^c^	
**Systolic blood pressure**	108.60 mmHg	7.17 mmHg	111.74 mmHg	8.03 mmHg	F(1, 580) = 16.306 *p* < 0.001 η_p_^2^ = 0.027	109.23 mmHg	7.41 mmHg	111.55 mmHg	8.31 mmHg	F(1, 220) = 4.837 *p* = 0.029 η_p_^2^ = 0.022
**Diastolic blood pressure**	67.24 mmHg	5.42 mmHg	69.15 mmHg	5.66 mmHg	F(1, 580) = 8.274 *p* = 0.004 η_p_^2^ = 0.014	67.52 mmHg	5.52 mmHg	69.17 mmHg	5.84 mmHg	F(1, 220) = 4.676 *p* = 0.032 η_p_^2^ = 0.021

^a^ ANCOVA calculations included participants from the entire study population; ^b^ ANOVA calculations included participants from the matched groups “users of OC” and “non-users of OC”. The groups were matched for the parameters age, body height, and SES; ^c^ standard deviation.

## Data Availability

The datasets presented in this article are not readily available because data cannot be shared publicly because there exist ethical restrictions. The LIFE Child study is a study collecting potentially sensitive information. Publishing data sets is not covered by the informed consent provided by the study participants. Furthermore, the data protection concept of LIFE requests that all (external as well as internal) researchers interested in accessing data sign a project agreement. Researchers that are interested in accessing and analyzing data collected in the LIFE Child study may contact the data use and access committee (dm@life.uni-leipzig.de). Requests to access the datasets should be directed to dm@life.uni-leipzig.de.
